# Novel human *CRYGD* rare variant in a Brazilian family with congenital cataract

**Published:** 2011-08-16

**Authors:** Eugênio Santana de Figueirêdo, Gabriel Gorgone Giordano, Anderson Tavares, Márcio José da Silva, José Paulo Cabral de Vasconcellos, Carlos Eduardo Leite Arieta, Mônica Barbosa de Melo

**Affiliations:** 1 Department of Ophthalmology, Faculty of Medical Sciences, University of Campinas - UNICAMP, Campinas, São Paulo, Brazil; 2 Laboratory of Human Molecular Genetics, Center of Molecular Biology and Genetic Engineering (CBMEG), University of Campinas – UNICAMP, Campinas, São Paulo, Brazil

## Abstract

**Purpose:**

To describe a novel polymorphism in the γD-crystallin (*CRYGD*) gene in a Brazilian family with congenital cataract.

**Methods:**

A Brazilian four-generation family was analyzed. The proband had bilateral lamellar cataract and the phenotypes were classified by slit lamp examination. Genomic DNA was extracted from peripheral blood and coding regions and intron/exon boundaries of the αA-crystallin (*CRYAA*), γC-crystallin (*CRYGC*), and *CRYGD* genes were amplified by polymerase chain reaction and directly sequenced.

**Results:**

Sequencing of the coding regions of *CRYGD* showed the presence of a heterozygous A→G transversion at c.401 position, which results in the substitution of a tyrosine to a cysteine (Y134C). The polymorphism was identified in three individuals, two affected and one unaffected.

**Conclusions:**

A novel rare variant in *CRYGD* (Y134C) was detected in a Brazilian family with congenital cataract. Because there is no segregation between the substitution and the phenotypes in this family, other genetic alterations are likely to be present.

## Introduction

Congenital cataract is characterized by the presence of an opacification of the lens at birth or during the first decade of life [1-5]. If untreated, it can result in significant visual impairment and even blindness [1]. This condition remains a leading cause of reversible childhood blindness in the world [4].

There are several causes for congenital cataract including metabolic disorders, infections during embryogenesis, gene defects, and chromosomal abnormalities [4]. About 8%−25% of congenital cataracts have hereditary etiology [4,6]. The most common mode of inheritance is autosomal dominant with high penetrance [4,7,8]. Inherited congenital cataracts exhibit a high interfamilial and intrafamilial phenotypic variability with a significant genotypic heterogeneity [1,4].

The water-soluble lens crystallin proteins (α-, β-, and γ-crystallins) account for approximately 90% of the total lens proteins and perform an important function in maintaining the transparency of the lens [1,4,7,9-11]. α-Crystallins exhibit chaperone-like activity and are present in high concentrations in the lens [4,11]. β- and γ-Crystallins have two domains composed of two highly stable protein structures called “Greek key” motifs [1,4,12]. The presence of α- and β-/γ-crystallins within the lens makes their encoding genes excellent candidates for congenital cataract, with several mutations already described [1,4,11].

In the present study, the analysis of the αA-crystallin (*CRYAA*), γC-crystallin (*CRYGC*), and γD-crystallin (*CRYGD*) genes was performed in a large Brazilian family presenting congenital cataract with phenotypic variability and suggestive high genetic heterogeneity.

## Methods

The study protocols adhered to the tenets of the Declaration of Helsinki and were approved by the Research Ethics Committees of the Faculty of Medical Sciences of the University of Campinas (Campinas, SP, Brazil). Appropriate informed consent from each participant was obtained.

### Patients and clinical data

A Brazilian family of four generations (8 affected members and 30 unaffected members) was investigated at the University of Campinas Ophthalmology Department. Affected status was determined by ophthalmic examination that included visual function, slit lamp examination, measurement of intraocular pressure, and fundus evaluation with dilated pupil. Cataract was classified based on its characteristics present at slit lamp evaluation or by the analysis of patients’ records who presented a history of cataract extraction. Detailed ocular, medical, and family histories were obtained from each available family member. Individuals had no suggestive history of intrauterine infection, unilateral cataract, and other ocular or systemic disorders. Fifty subjects without diagnostic features of congenital cataract were recruited as normal controls, representing a sample of the Brazilian population.

### Genomic DNA preparation and molecular analysis

Venous blood (5–10 ml) was collected for genomic DNA extraction and subsequent molecular genetic analysis. Polymerase chain reaction (PCR) was used to amplify all the exons and intron/exon boundaries of the candidate genes (*CRYAA, CRYGC* and *CRYGD*). The PCR cycling conditions were: initial denaturation at 94 °C for 5 min followed by 35 cycles of 94 °C for 1 min, X °C for 1 min, 72 °C for 1 min, and final extension at 72 °C for 7 min. Amplification of samples was performed in a “Mastercycler EP Gradient S” thermalcycler (Eppendorf, Hamburg, Germany). Oligonucleotide primer pairs, PCR product sizes, and annealing temperatures are described in Table 1. PCR products were electrophoresed in 1.5% agarose gels containing 0.05% ethidium bromide, purified, and submitted to direct sequencing on the ABI PRISM 3700 Genetic Analyzer automated sequencer (Applied Biosystems, Foster City, CA). The sequencing reactions conditions were: 1 cycle of 96 °C for 1 min 30 s and 24 cycles of 96 °C for 12 s, 50 °C for 6 s, and 60 °C for 4 min, using Big Dye Terminator Ready Reaction v3.1 (ABI PRISM Big Dye Terminator Cycle Sequencing Kit; Applied Biosystems). Sequencing results were analyzed through submission to similarity search using the “search algorithm” BLAST.

**Table 1:** Primers for PCR amplification of the *CRYAA*, *CRYGC*, and *CRYGD* genes, product sizes, and annealing temperatures.

**Gene**	**Exon**	**Strand**	**Sequence (5′→3′)**	**Fragment Size (bp)**	**T (°C)**
*CRYAA*	1	sense	CACGCCTTTCCAGAGAAATC	466	63.9
	1	antisense	CTCTGCAAGGGGATGAAGTG		
	2	sense	CTTGGTGTGTGGGAGAAGAGG	377	58.0
	2	antisense	TCCCTCTCCCAGGGTTGAAG		
	3	sense	CCCCCTTCTGCAGTCAGT	989	57.0
	3	antisense	GCTTGAGCTCAGGAGAAGGA		
*CRYGC*	1 - 2	sense	ACCAGAGAACAAGGACACAATC	674	66.6
	1 - 2	antisense	TGGCTTATTCAGTCTCTGATG		
	3	sense	ATTCCATGCCACAACCTACC	590	66.3
	3	antisense	CCAACGTCTGAGGCTTGTTC		
*CRYGD*	1 - 2	sense	CCCTTTTGTGCGGTTCTTG	596	54.0
	1 - 2	antisense	TTTGTCCACTCTCAGTTATTGTGAC		
	3	sense	TGTGCTCGGTAATGAGGAG	700	61.0
	3	antisense	AGGCCAGAGAATCAAATGAG		

### Computational methods

Algorithms available in the internet as automated methods were used as tools to evaluate the possible influence of the substitution of an amino acid in the protein function. Sorting Intolerant from Tolerant (SIFT) amino acid substitutions assumes that the important amino acids tend to be conserved between species. This method assigns a substitution probability from 0 to 1 for each possible amino acid change. Substitution with probabilities less than 0.05 are considered intolerant. Polymorphism Phenotyping 2 (PolyPhen-2) algorithm considers the conservation of amino acids between species and physicochemical differences between wild and mutant proteins. The Align-GVGD method is based on chemical differences, polarity and molecular volume between permuted amino acids. Scores range from 15 to 215, with scores >100 predictive of deleterious mutations and tolerable <60.

## Results

### Clinical evaluation

**Figure 1:**
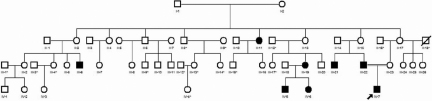
Pedigree of a four-generation family harboring congenital cataract. The proband is marked with an arrow. Squares and circles indicate males and females, respectively. Black and white symbols denote affected and unaffected individuals, respectively. A slash through the symbol means that the family member is deceased. Thirty-eight individuals (eight affected and thirty unaffected) from the family were enrolled and underwent ophthalmic examinations and genetic analysis (individuals marked by an asterisk did not participate in the study).

This family is represented by four generations of individuals. Four members were affected by bilateral lamellar cataract, including the proband (III-19, IV-5, IV-6, and IV-7). Four individuals had bilateral pulvurulent cataract (II-11, III-6, III-21, and III-22). The two oldest individuals of the family (I-1 and I-2) had nuclear and anterior cortical lens opacities with characteristics of senility. One patient with lamellar cataract underwent surgery at another hospital, presenting bilateral aphakia and severe amblyopia in his right eye. Three other individuals with lamellar cataract were operated at 1, 12, and 18 months of age, respectively (Figure 1). Visual acuity in this family subsequently to surgery ranged from count fingers to 1.0 (Table 2).

**Table 2:** Clinical evaluation of patients affected by congenital cataract.

**Individual**	**Diagnostic Age (years)**	**Phenotype**	**Surgery**	**BCVA OD**	**BVCA OS**	**Polymorphism Y134C in *CRYGD***
II-11	46	Pulvurulent	No	1.0	1.0	No
III-6	24	Pulvurulent	No	1.0	1.0	No
III-19	at birth	Lamellar	Yes	CF	0.6	No
III-21	26	Pulvurulent	No	1.0	1.0	No
III-22	25	Pulvurulent	No	1.0	1.0	Yes
IV-5	at birth	Lamellar	Yes	0.4	0.5	No
IV-6	at birth	Lamellar	Yes	0.05	0.05	No
IV-7	at birth	Lamellar	Yes	0.4	0.3	Yes

BCVA OD: Best corrected visual acuity in right eye. BCVA OS: Best corrected visual acuity in left eye. CF: Count fingers.

### Mutation analysis

**Figure 2:**
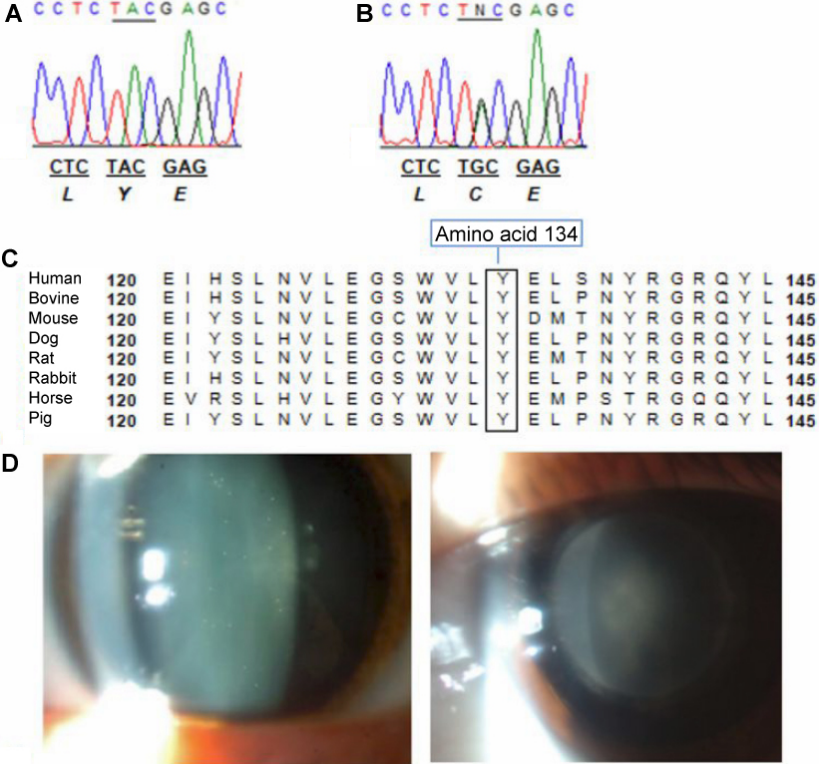
Sequence analysis of *CRYGD*. **A**: Sequence of a member without the polymorphism. **B**: Heterozygous polymorphism detected in exon 3 of *CRYGD* (individuals II-15, III-22, and IV-7). **C**: Multiple sequence alignment of the CRYGD protein (codons 120–145) in different species, demonstrating that residue 134 is highly conserved. **D**: Slit-lamp photograph, showing pulvurulent cataract in individual III-22 (left) and lamellar cataract in individual IV-7 (right).

Direct sequencing was performed to cover exons and intron-exon boundaries of *CRYAA, CRYGC*, and *CRYGD*. A heterozygous A→G transition was identified at c.401 position of exon 3 in two affected members (III-22 and IV-7) and in one unaffected member (II-15; Figure 2). This alteration resulted in the missense substitution of a wild type tyrosine to a cysteine (Y134C) in the CRYGD protein. The variant was completely absent in 100 chromosomes of 50 unrelated controls. The other affected members showed no alterations in any of the evaluated crystallins.

### Computational method analysis

The SIFT method revealed a score of 0.00 to position Y134. The PolyPhen-2 algorithm showed a score of 1.000 and the Align-GVGD method presented a score of 193.72.

## Discussion

A considerable amount of mutations associated with human congenital cataracts have been described in *CRYGD* [1,4,13]. These mutations alter the stability and/or the solubility of γ-crystallins and contribute to loss of transparency of the lens fibers [1]. Therefore, several crystallin genes have been considered as candidates for hereditary congenital cataract [4,6].

The autosomal dominant congenital cataract has a high penetrance and is usually symmetric in affected individuals. However, there may be considerable variability within a pedigree and between the two lenses of the same individual [14-21]. This may explain the different phenotypes observed in the family evaluated.

There are few studies about genetic alterations related to congenital cataract in the Brazilian population. Santana et al. [4] related the Y56X substitution in exon 2 of *CRYGD* in a Brazilian family with the nuclear phenotype, resulting in a truncated protein missing 118 amino acids. In the same study, a mutation in exon 1 of *CRYAA* (R12C) was reported.

This study describes the Y134C substitution, located in exon 3 of *CRYGD*, which, to our knowledge, was not reported in any population to date. The effect of this amino acid substitution was evaluated through Polyphen-2, SIFT, and Align-GVGD computational programs. The results of in silico analysis of these algorithms suggest that a variation Y134C in *CRYGD* tends to be intolerant as evaluated by the SIFT method that revealed a score of 0.00 to position Y134. Furthermore, PolyPhen-2 achieved a score of 1.000, showing that the amino acid tyrosine at this position is highly conserved among species (Figure 2) as well as the physicochemical differences between wild and mutant proteins indicate “probably damaging.” Finally, the Align-GVGD method presented a score of 193.72, demonstrating that the substitution is probably deleterious as well. Santana and colleagues [4] used these same algorithms to analyze the R12C mutation in *CRYAA* which was segregating with congenital cataract in a Brazilian family. They found similar scores to those obtained in this study.

The other mutation described by Santana and colleagues [4], the Y56X in *CRYGD*, does not apply for the three in silico methods due to generation of a truncated protein. Chan et al. [22] reinforced that when the results of these algorithms are combined, their isolated predictive value is increased.

Besides the analysis of these three algorithms suggesting that the Y134C variant in *CRYGD* could be a causative disease mutation and that it occurred in a gene already associated with congenital cataract, the evaluation of its segregation pattern within this family is not consistent with its relation to the disease. The presence of this alteration in individuals II-15, III-22, and IV-7 and its absence in the other family members might indicate the occurrence of a de novo mutation. The fact that individual III-21 is affected but does not have the same variation as his brother as well as the presence of congenital cataract in other family members without this variation (II-11, III-6, III-19, IV-5, and IV-6) indicates that other gene is involved in the etiology and that the Y134C variant is a rare SNP probably not associated with the disease.

There are six affected individuals who do not present the substitution. Santana et al. [4] found a novel polymorphism in *CRYGC* (S119S) in a family with bilateral autosomal dominant congenital nuclear cataract and microcornea that was absent in some affected individuals. It was suggested that this absence could be justified by recombination events and that this polymorphism could be a marker of an unidentified gene located in this region.

Isolated autosomal dominant cataract is genetically heterogeneous and to date 14 genes and nine additional loci have been implicated with the disease [11,23]. The manifestation of the disease in this family may be influenced by more than one gene with incomplete penetrance. Additionally,the possible interaction with other genes, not analyzed in this study, may have interfered with the Y134C substitution in the susceptibility to congenital cataract development.

In conclusion, we found a novel substitution in *CRYGD* in a Brazilian family with congenital cataract in which there is no accurate segregation with the disease, hence, it is probably not a disease causing mutation. Further studies in this family involving structural and functional genes associated with congenital cataract are necessary to better understand the mechanisms underlying congenital cataract development.
